# Accumulation of Intrahepatic TNF-α-Producing NKp44^+^ NK Cells Correlates With Liver Fibrosis and Viral Load in Chronic HCV Infection

**DOI:** 10.1097/MD.0000000000003678

**Published:** 2016-05-13

**Authors:** Isabelle Nel, Olivier Lucar, Caroline Petitdemange, Vivien Béziat, Martine Lapalus, Pierre Bédossa, Patrice Debré, Tarik Asselah, Patrick Marcellin, Vincent Vieillard

**Affiliations:** From the Sorbonne Universités (IN, OL, CP, VB, PD, VV), UPMC Univ Paris 06, INSERM U1135, CNRS ERL8255, Centre d’Immunologie et des Maladies Infectieuses (CIMI-Paris), Paris; Univ Paris Diderot (ML), INSERM UMR 1149, CRI Paris Montmartre, Clichy; AP-HP (PB), Service d’Anatomie Pathologique; and AP-HP (TA, PM), Service d’Hépatologie, Hôpital Beaujon, Clichy, France.

## Abstract

In the setting of chronic hepatitis C virus (HCV) infection, changes in natural killer (NK) cells have been shown to reflect activation in response to virus stimulation. The contribution of individual natural cytotoxicity receptors to HCV infection remains to be clarified. NKp44 is the sole specific natural cytotoxicity receptor expressed only on activated NK cells.

In this study, peripheral blood and liver NK-cell subsets were purified from 31 patients with chronic C hepatitis or nonalcoholic steatohepatitis, and then characterized by flow cytometry. Their polyfunctional activity was determined by expression of the CD107a degranulation marker, together with intracellular cytokine production.

Unlike the patients with nonalcoholic steatohepatitis, patients with chronic HCV infection had a higher frequency of NKp44^+^ NK cells in the liver than in their peripheral blood (*P* < 0.0001). Intrahepatic NKp44^+^ NK cells from HCV^+^ individuals produced higher levels of tumor necrosis factor-α than did NKp44^−^ NK cells (*P* = 0.0011). Importantly, the frequency of intrahepatic NKp44^+^ NK cells was correlated with both HCV-RNA levels (*P* = 0.0234) and stage of fibrosis (*P* = 0.0003).

Our findings suggest that the accumulation of intrahepatic tumor necrosis factor-α-producing NKp44^+^ resident NK cells play a role in the liver damage associated with chronic HCV infection.

## Abstract 2

Supplemental Digital Content is available in the text

## INTRODUCTION

Approximately 170 million people worldwide are infected with hepatitis C virus (HCV), many of them diagnosed only after decades of chronic infection. Only a minority of patients can clear the virus spontaneously during acute infection. HCV remains a leading cause of liver cirrhosis and hepatocellular carcinoma, despite great progress in the development of direct-acting antivirals (DAAs) able to cure more than 90% of HCV genotype 1-infected patients after 12 weeks of treatment.^1^ The mechanisms associated with both the failure of viral clearance and the development of chronic infection are not yet well understood. It has, however, been observed that most patients are unable to clear the virus and thus develop viral persistence.^1^ Natural killer (NK) cells are involved in every stage of HCV infection. As a major component of the intrahepatic lymphocyte pool, they are likely to be important sentinels that survey the liver for signs of damage or cellular stress. Accordingly, HCV must subvert NK cell-mediated responses to establish persistent infection.^2–6^ NK cells participate in viral clearance by killing infected cells directly, through the release of cytotoxic molecules, such as perforin and granzymes, and by producing cytokines such as interferon (IFN)-γ and tumor necrosis factor (TNF)-α. Under normal immune surveillance, NK cells express inhibitory receptors, including killer Ig-like receptors (KIR), Ig-like transcript 2, and the CD94:NKG2A heterodimer, which recognize Major histocompatibility complex class I molecules as their cognate ligands. Cytotoxicity, however, occurs when stimulatory signals outweigh HLA-I inhibition. Several of these activating receptors have been characterized, including NKG2C, NKG2D, and the natural cytotoxicity receptors (NCRs): NKp30, NKp44, and NKp46.^7–9^ Compared with the constitutively expressed NKp30 and NKp46 receptors, NKp44 is especially interesting because it is induced only after cell activation.^10^ NKp44 triggers NK-cell cytotoxicity upon recognition of a new mixed lineage leukemia 5 (MLL5) isoform, expressed only on stressed cells.^11,12^ Several “pathogenic” ligands for NKp44 that stimulate NK-cell response have been described, including influenza virus hemagglutinin and viral Hemagglutinin-neuraminidase.^9^ NKp44 also interacts with the E glycoprotein of the dengue and West Nile viruses.^13^

Several lines of evidence indicate that NK cells play a role in acute HCV infection. Khakoo et al^14^ reported that individuals homozygous for the genes encoding KIR2DL3, an inhibitory NK cell receptor, and its ligand are more likely than others to resolve infection by HCV, consistent with a subsequent finding that KIR2DL3^+^NKG2A^−^ NK cells seem to mediate early control of HCV infection, before seroconversion.^15^ Furthermore, several studies over the past decade have reported that chronic HCV infection is characterized by functionally and phenotypically impaired NK-cell responses that may contribute to the progression of liver disease^1,3,5,16,17^ and are partially restored during antiviral therapy.^18^ The scarcity of data about intrahepatic NK cells and their discrepancy concerning NKp44 in patients with chronic HCV infection^19,20^ prompted us to compare peripheral blood and liver NK cells from these patients to evaluate the role of intrahepatic NKp44^+^ NK cells in the immunopathogenesis of this disease.

## METHODS

### Patient Populations

This study included 31 patients scheduled for liver biopsy at Beaujon Hospital (Clichy, France) from December 2009 through February 2014: 21 with chronic HCV infection and 10 with nonalcoholic steatohepatitis (NASH) (Table 1). The study protocol conformed to the ethical guidelines of the 1975 Declaration of Helsinki and was approved by the Comité de Protection des Personnes (CCP), Ile de France IV (# 2009/19NI). All patients provided written informed consent. Infected patients were included if they had chronic HCV infection, defined by seropositivity for HCV-RNA, respectively, for at least 12 months. Coinfected patients, patients on antiviral treatment, and patients with liver transplants were excluded. Staging and grading of pathology were determined as part of the hospital's routine diagnostic work-up. An experienced pathologist, blinded to the clinical and laboratory data, analyzed the liver biopsy specimens according to the Metavir scoring system: necroinflammatory activity (A) was graded as A0 (absent), A1 (mild), A2 (moderate), or A3 (severe). Fibrosis stage (F) was scored as F0 (absent), F1 (portal fibrosis), F2 (portal fibrosis with >1 septum), F3 (septal fibrosis), or F4 (cirrhosis).^21^

**TABLE 1:**
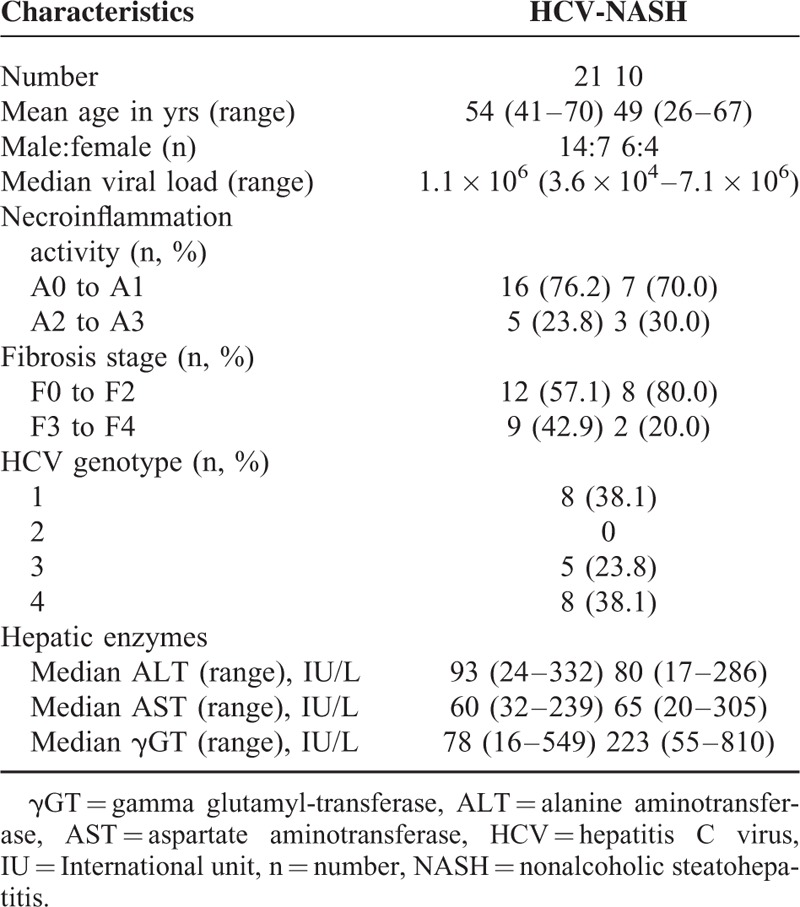
Characteristics of Subjects Enrolled in This Study

### Sample Preparation

Peripheral blood samples were collected in heparin tubes the same day as the biopsy. Liver biopsies were prepared as previously described.^11,22^ Briefly, they were mechanically dissociated through a 70-μm cell strainer (BD Pharmingen), before incubation with 10 mg/mL collagenase II-S (Sigma-Aldrich) in medium containing trypsin inhibitor (Sigma-Aldich) and DNase I (Roche Life Science) for 1.5 hours at 37°C on a rotary shaker. After additional washes, the suspension was filtered through a 40-μm cell strainer, centrifuged, and used directly for phenotypic and/or functional assays analyzed by flow cytometry.

### Flow Cytometric Analysis

Natural killer cells were analyzed after staining with an appropriate monoclonal antibody (mAb) cocktail, as previously described^22,23^: anti-CD45 (#J33), anti-CD3 (#UCHT1), anti-CD56 (#N901), anti-CD16 (#3G8), anti-CD159a/NKG2A (#Z199), CD335/NKp46 (#BAB281), anti-CD336/NKp44 (#Z231), anti-CD69 (#FN50), anti-Cx3CR1 (#2A9–1), and anti-CD127 (#R34.34) mAbs from Coulter; anti-CD62L (#DREG-56; BD Pharmingen), anti-CD337/NKp30 (#AF29–4D12), and anti-NKG2C (#134591) mAbs from R&D systems, and also a cocktail of anti-panKIR-L including anti-KIR2DL1 (#143211) and anti-KIR3DL1 (#177407); and anti-KIR2DL2/KIR2DL3 (#DX27; Miltenyi Biotech) mAbs. Populations of interest within the CD45^+^ lymphocytic population were gated on CD3^−^CD56^+^ NK cells. At least 20,000 CD45^+^ cells or 1000 CD3^-^CD56^+^ NK cells were acquired on a Gallios flow cytometer (Beckman Coulter) and then analyzed with Flow Jo version 9 (TreeStar) (Supplementary Figure 1), as described.^22^

### Intracellular Cytokine Production and Polyfunctionality Assay

Functional capacity of NKp44^+^ NK cells compartment has been tested in 10 samples from peripheral blood and liver biopsies. Cells were incubated overnight with 10 ng/mL interleukin (IL)-12 and 100 ng/mL IL-18 at 37°C and 5% CO_2_. They were thereafter incubated for 5 hours in the presence of Golgi Stop and Golgi Plug solutions (BD Biosciences), and then stained with cell surface markers (anti-CD3, anti-CD56, and anti-NKp44 mAbs). Next they were fixed, permeabilized with a cytofix/cytoperm kit (Becton Dickinson), and then stained intracellularly with anti-IFN-γ (#B27; Becton Dickinson) and anti-TNF-α (#Mab11; eBiosciences) mAbs, as described.^23^

For the polyfunctionality assays, nonstimulated NK cells were incubated in the presence of the standard K562 target cells (ATCC CCL243) and hepatocellular carcinoma Hep G2 cells, kindly provided by Professor D. Mazier (CIMI-Paris, Paris, France), at an effector:target (E:T) cell ratio of 1:1 in the presence of an anti-CD107a mAb (#H4A3; Becton Dickinson), used as a degranulation cell-surface marker, as described.^22,23^ Data were analyzed with Flow Jo version 9 (TreeStar), and the rates of NK cells positive for 0, 1, 2, or 3 functions were defined with this software's “Boolean gate” algorithm. Pie charts, generated with Spice software (NIAI freeware),^24^ present the frequency of NK cells positive for 0, 1, 2, or 3 responses (to CD107a, IFN-γ, and/or TNF-α). Arcs depict the frequency of cells positive for each of CD107a, IFN-γ, and TNF-α.

### Quantification of Cytokines

Cytokine analysis was performed in the plasma of the 21 HCV^+^ patients, with Quantikine IFN-γ and TNF-α immunoassays (R&D Systems), according to the manufacturer's protocols. Frozen plasma samples (preserved at −80 °C) were used for testing after clarification by centrifugation (30 min at 1000*g*) at 4°C. Experiments were performed in duplicates. The sensitivity was 8.0 pg/mL for IFN-γ and 1.6 pg/mL for TNF-α.

### Statistical Analysis

Statistical analyses were performed with Prism-5 software (GraphPad Software). The nonparametric “Wilcoxon rank test” was used to compare 2 related samples. Statistical significance was defined by a *P* value less than 0.05 with a 2-tailed test. Nonparametric correlations were assessed by determining Spearman rank correlation coefficient.

## RESULTS

### Accumulation of Intrahepatic NKp44^+^ NK Cells in Chronic HCV-infected Patients

**FIGURE 1:**
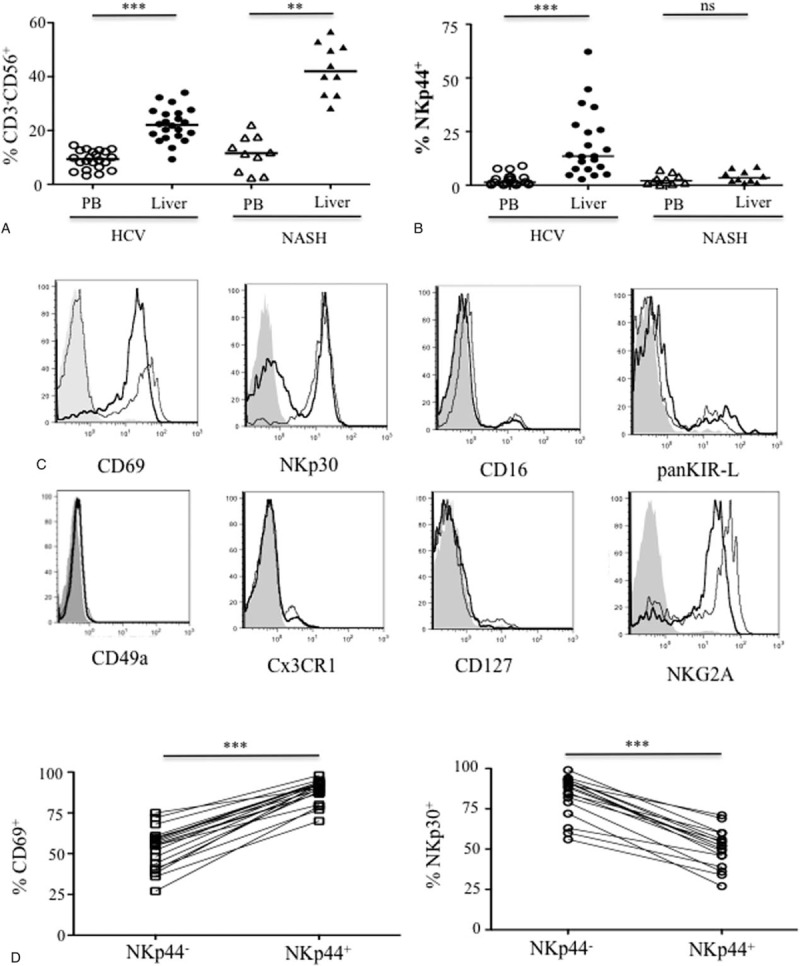
Accumulation of intrahepatic NKp44^+^ NK cells in chronically HCV-infected patients. Flow cytometry analysis of NK cells from patients with HCV (n = 21) or NASH (n = 10) (Table 1). A, Frequency of peripheral blood (PB) and intrahepatic (liver) CD3^−^CD56^+^ NK cells. B, Expression of NKp44 gated on CD3^−^CD56^+^ cells in peripheral blood mononuclear cells (PBMC) and liver biopsies. Statistical analysis used the Wilcoxon rank test to compare paired blood and intrahepatic samples. (∗∗) *P* < 0.01; (∗∗∗) *P* < 0.001. C, Representative phenotypic characterization of intrahepatic NKp44^+^ NK cells (bold black line), compared with NKp44^−^ cells (light black line) and isotype controls (gray panel). D, Frequency of CD69^+^ (left panel) and NKp30^+^ (right panel) within intrahepatic NKp44^−^ and NKp44^+^ NK-cell subsets from the 21 HCV-infected patients. HCV = hepatitis C virus, NASH = nonalcoholic steatohepatitis, NK = natural killer, ns = nonsignificant.

Previous reports published on the properties of intrahepatic NK cells in chronic HCV-infected patients are conflicting, likely related to limited cell numbers from biopsies or different gating strategies by flow cytometry.^3,16–18,25,26^ In this study, we observed that the overall frequency of NK cells was significantly higher in the liver than in the peripheral blood in both HCV-infected patients and patients with NASH (Figure 1A). However, in contrast to the patients with NASH, HCV^+^ patients showed a significant accumulation of NKp44^+^ CD3^−^CD56^+^ cells in the liver, compared with their blood samples (*P* < 0.0001, median 13.5%; Figure 1B).

This observation prompted us to characterize the intrahepatic NKp44^+^ cells from HCV^+^ patients in more detail. They did not express either CD127 (IL7Rα) or CD49a, which indicates that they are distinct from both the NKp44^+^ invariant lymphocyte cells (ILC1) in tonsils^27^ and the liver-resident CD49a^+^ NK-cell subset (Figure 1C), consistent with the findings of Marquardt et al.^28^ Furthermore, compared with peripheral NK cells, they did not express high frequencies of CD16, panKIR-L, CD62L, or Cx3CR1, which is associated with the migration of circulating CD56^dim^ NK cells to sites of inflammation (Figure 1C and Supplementary Figure 2 ). Although a high frequency of NKp44^+^ cells expressed NKG2A, its mean fluorescence intensity (MFI) was slightly lower than in the NKp44^−^ cells (Figure 1C and Supplementary Figure 2). NKp44^+^ NK cells had a significantly higher frequency of activation receptor CD69 than did the NKp44^-^ cells (*P* < 0.0001; Figure 1D), and a lower frequency of NKp30 (*P* = 0.0003; Figure 1D). Instead, the intrahepatic NKp44^+^ cells displayed the profile of activated CD3^−^CD56^bright^CD16^−^NKp30^low^ NK cells.

### Intrahepatic NKp44^+^ NK Cells are Correlated With HCV Viral Load and Fibrosis Activity

**FIGURE 2:**
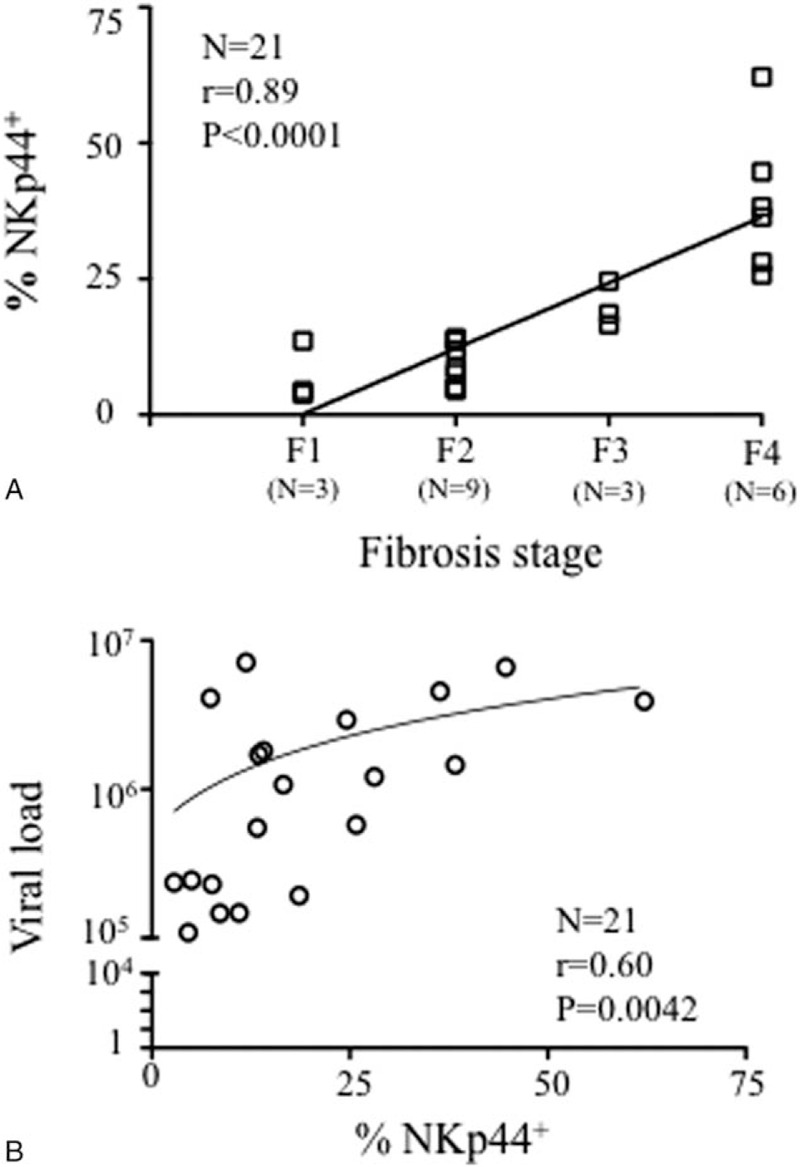
Correlation between intrahepatic NKp44^+^ NK cells, virological and clinical parameters in chronically HCV-infected patients (n = 21). Linear regression between the frequency of intrahepatic NKp44^+^ NK cells and the fibrosis stage (F1–F4) (A) or with viral load (B). The Spearman test showed significant statistical correlations. HCV = hepatitis C virus, NK = natural killer.

Next, we attempted to identify any statistical correlations between the accumulation of intrahepatic NKp44^+^ cells and biochemical (serum alanine aminotransferase, aspartate aminotransferase, and gamma glutamyl-transferase), virologic (HCV genotype, HCV-RNA), and/or clinical (necroinflammatory activity and fibrosis stage) parameters. No association was found with HCV genotype, hepatic enzyme levels, or necroinflammatory activity (data not shown). However, the frequency of intrahepatic NKp44^+^ cells was significantly correlated with the liver fibrosis stage (*P* < 0.0001, *r* = 0.89; Figure 2A) and, to a lesser extent, with viral load (*P* = 0.042, *r* = 0.60; Figure 2B).

### Intrahepatic NKp44^+^ NK cells Produce High Levels of TNF-α in Association With HCV Viral Load and Fibrosis Activity

**FIGURE 3:**
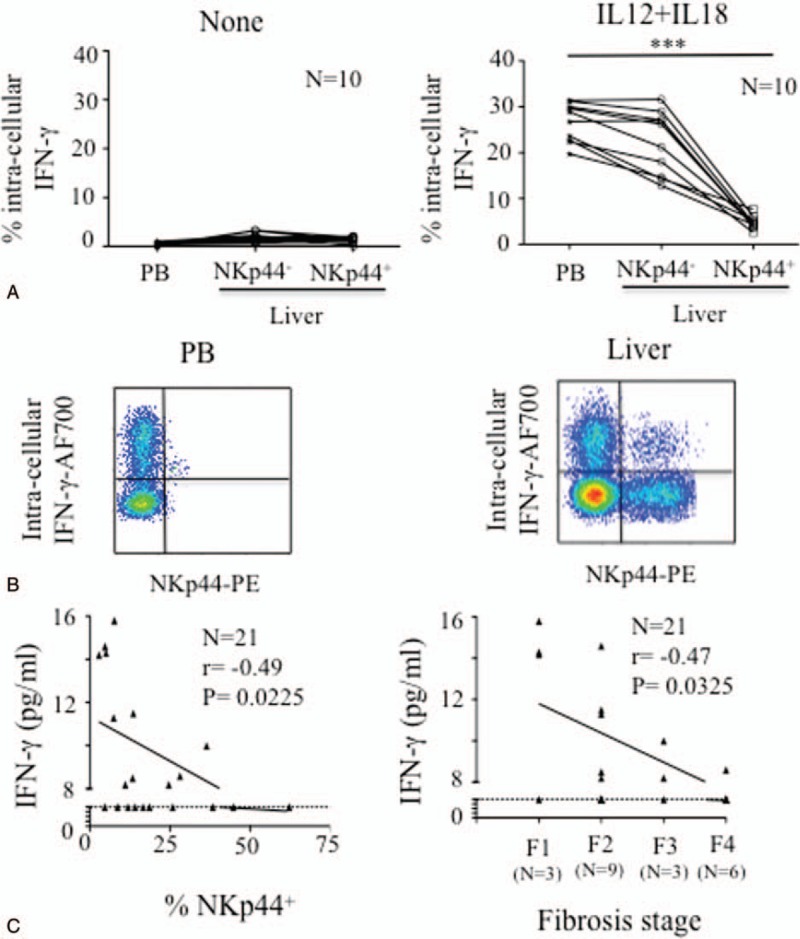
Lower level of IFN-γ in intrahepatic NKp44^+^ NK cells from HCV-infected patients than in its NKp44^−^ counterparts or in peripheral blood cells. A, Intracellular production of IFN-γ in peripheral blood (PB) and intrahepatic (liver) NKp44^−^ or NKp44^+^ CD3^−^CD56^+^ NK cells before (none) or after overnight stimulation with IL-12 plus IL-18. Samples from 10 of the 13 patients with a frequency of NKp44^+^ cells superior to the median (>13.5%) were tested. Statistical correlation used 1-way analysis of variance with Tukey multiple comparison tests. B, Representative HCV^+^ sample for intracellular production of IFN-γ in regard of NKp44 expression, gated on CD3^−^CD56^+^ NK cells from peripheral blood (PB) and liver biopsy (liver), after overnight stimulation with IL-12 plus IL-18. C, Correlation of the plasma level of IFN-γ with the frequency of intrahepatic NKp44^+^ NK cells and the fibrosis stage in the 21 HCV-infected patients. The Spearman test showed significant statistical correlations. Dotted lines indicate the sensitivity of IFN-γ detection. HCV = hepatitis C virus, IFN-γ = interferon-gamma, NK = natural killer.

We next assessed the overall functional ability of NKp44^+^ NK cells within the intrahepatic compartment in 10 of the 13 liver biopsies, with an NKp44 frequency greater than the median (13.5%) (13.6%–62.2%) (Figure 1B); the 3 last biopsies were too small size to undergo functional analyses. One of the main functions of NK cells is the production and release of IFN-γ and TNF-α. After treatment with IL-12 and IL-18, the frequency of NK cells able to produce IFN-γ was lower in the intrahepatic NKp44^+^ cells than in the other subsets in the 10 tested liver biopsies (Figure 3A and B). Consistently with a previous study,^29^ the plasma level of IFN-γ, in the plasma of the 21 HCV^+^ patients, was inversely correlated with the stage of fibrosis (*P* = 0.0325, *r* = −0.47), and also with the frequency of intrahepatic NKp44^+^ NK cells (*P* = 0.0225, *r* = −0.50; Figure 3C). Neither intracytoplasmic nor plasma levels of IFN-γ were correlated with viral load (data not shown).

**FIGURE 4:**
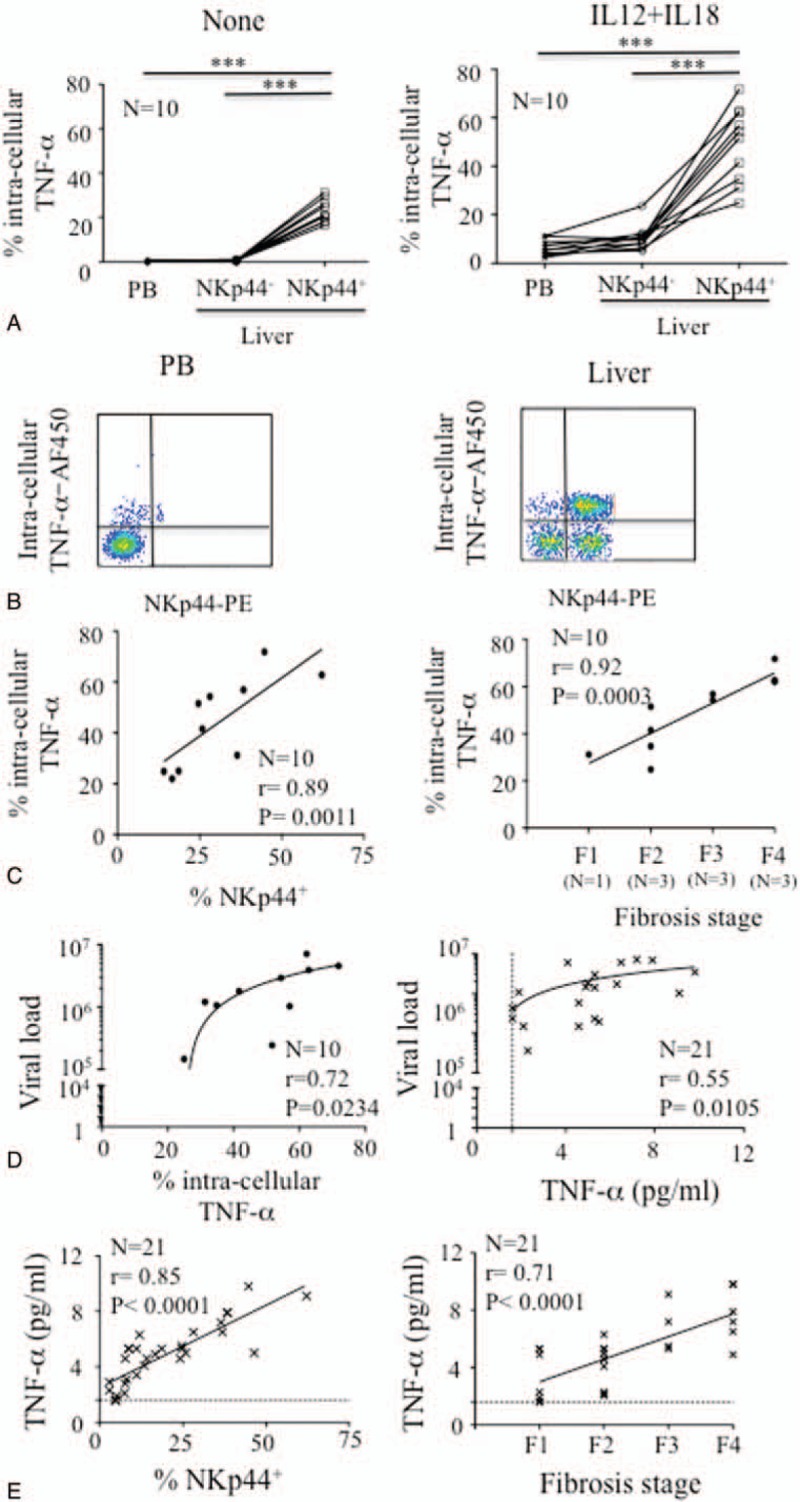
Overproduction of TNF-α by intrahepatic NKp44^+^ NK cells in chronically HCV-infected patients. A, Intracellular production of TNF-α in peripheral blood (PB) and intrahepatic (liver) NKp44^−^ or NKp44^+^ CD3^−^CD56^+^ NK cells before (none) or after overnight stimulation with IL-12 plus IL-18. Samples from 10 of the 13 patients with a frequency of NKp44^+^ cells superior to the median (>13.5%) were tested. Statistical correlation used the 1-way analysis of variance with Tukey multiple comparison tests. B, Representative HCV^+^ sample for intracellular production of TNF-α in regard of NKp44 expression, gated on CD3^−^CD56^+^ NK cells from peripheral blood (PB) and liver biopsy (liver), after overnight stimulation with IL-12 plus IL-18. C, Correlation between the intracellular production of TNF-α in intrahepatic NK cells and the frequency of intrahepatic NKp44^+^ NK cells or fibrosis stages. Samples from 10 of the 13 patients with a frequency of NKp44^+^ cells superior to the median (>13.5%) were tested. The Spearman test showed significant statistical correlations. D, Correlation between the intracellular production (n = 10; left panel) or plasma level (n = 21; right panel) of TNF-α and viral load in 10 patients with a frequency of NKp44^+^ cells superior to the median (>13.5%). Significant statistical correlations were obtained using the Spearman test. E, Correlation between the plasma level of TNF-α, the frequency of intrahepatic NKp44^+^ NK cells, and fibrosis stage in the 21 HCV-infected patients. The Spearman test showed significant statistical correlations. Dotted lines indicate the sensitivity of TNF-α detection. HCV = hepatitis C virus, IL = interleukin, NASH = nonalcoholic steatohepatitis, NK = natural killer, TNF-α = tumor necrosis factor-alpha.

In contrast to IFN-γ, the intracellular level of TNF-α was significantly higher in intrahepatic NKp44^+^ NK cells than in either their NKp44^−^ counterparts or the peripheral blood NK cells, both before and after treatment with IL-12 plus IL-18, in the 10 tested liver biopsies (Figure 4A and B). The frequency of NK cells producing TNF-α correlated with the frequency of intrahepatic NKp44^+^ NK cells (*P* = 0.0011, *r* = 0.89; Figure 4C). More importantly, NKp44^+^ NK cells producing TNF-α were significantly associated with the stage of fibrosis (*P* = 0.0003, *r* = 0.92; Figure 4C) and viral load (*P* = 0.0234, *r* = 0.72; Figure 4D). These data are consistent with the correlation of the TNF-α plasma level with the frequency of intrahepatic NK cells, in the plasma of the 21 HCV^+^ patients (*P* = 0.0388, *r* = 0.46; Supplementary Figure 3) and still more specifically with the NKp44^+^ cell subset (*P* < 0.0001, *r* = 0.85; Figure 4E). The TNF-α plasma level was also correlated with fibrosis stage (*P* < 0.0001, *r* = 0.71; Figure 4E) and viral load (*P* = 0.0105, *r* = 0.55; Figure 4D). Of note, the plasma levels of TNF-α and IFN-γ were not correlated with any other laboratory or clinical parameters (data not shown).

**FIGURE 5:**
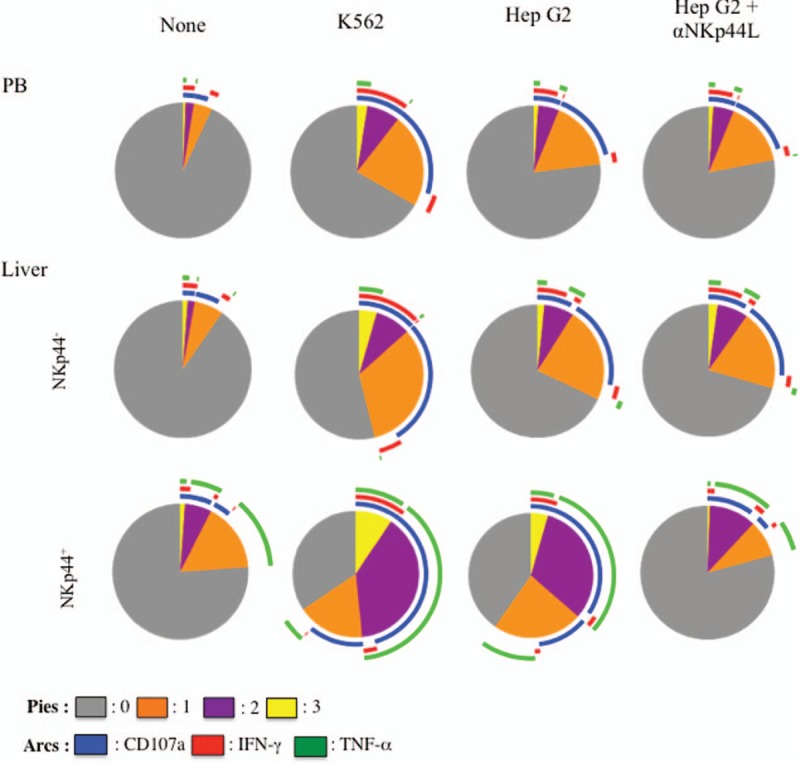
Polyfunctional activity of intrahepatic NKp44^+^ NK cells in chronically HCV-infected patients. Assays were performed with peripheral blood CD3^−^CD56^+^ NK cells (PB) and NK cells from liver biopsy samples (NKp44^−^ and NKp44^+^ NK-cell subsets) from the same HCV-infected patients. Pie chart representation of the polyfunctional activity representing the cell-surface expression of CD107a, and intracellular expression of IFN-γ and TNF-α assessed in the absence (none) or presence of K562, Hep G2. Data are the means of the 10 available samples with a frequency of NKp44^+^ cells superior to the median (>13.5%). Hep G2 were treated with 10 μg/mL of anti-NKp44L (aNKp44L) (right panel) or with IgM isotype control (not shown), as described.^11^ The Boolean gate function of FlowJo software was used to determine whether cells expressed 0 (gray), 1 (orange), 2 (purple), or 3 (yellow) functions simultaneously (pie charts). Colored arcs represent the frequency of NK cells expressing CD107a (blue), IFN-γ (red), and TNF-α (green). HCV = hepatitis C virus, NASH = nonalcoholic steatohepatitis, NK = natural killer, PB = peripheral blood, TNF-α = tumor necrosis factor-alpha.

We next attempted to determine the capacity of intrahepatic NKp44^+^ NK cells to simultaneously degranulate and produce cytokines (IFN-γ and/or TNF-α). This polyfunctional assay was performed against standard K562 target cells and Hep G2 hepatocellular cells—2 cell lines expressing NKp44L (Supplementary Figure 3), the cellular ligand of NKp44. Stimulation with both targets produced similar functional profiles in peripheral blood and intrahepatic NKp44^−^ NK-cell subsets, although the level of polyfunctionality was slightly lower with Hep G2 (Figure 5). Intrahepatic NKp44^+^ NK cells were more polyfunctional, than liver and peripheral NKp44^−^ cells, with a strong capacity to simultaneously degranulate and produce TNF-α (Figure 5); this capacity was lower in the presence of Hep G2 than K562 target cells, in accordance with their respective cell surface expression of NKp44L (Supplementary Figure 4).

Further proof that NKp44 is involved in the NK cytotoxicity mediated by the intrahepatic NK cells came from the pretreatment of Hep G2 cells by anti-NKp44L mAb. The presence of anti-NKp44L mAb strongly inhibited the polyfunctional activity of intrahepatic NKp44^+^ NK cells (by up to 55% for their capacity to perform at least 1 function), whereas the polyfunctionality of their NKp44^-^ counterparts remained similar to that observed in untreated Hep G2 cells (Figure 5, right panel). These data suggest that NKp44 expressed by intrahepatic NK cells could be involved in the destruction of NKp44L-expressing hepatocytes.

We then considered whether intrahepatic NKp44^+^ NK cells were able to interfere with hepatic stellate cells (HSCs), which are considered the main contributors to liver fibrosis.^30^ NKp44L expression was tested on the LX-2 human HSC cell line, which retains key features of primary HSCs.^2,31^ The failure of the LX-2 cells, unlike the Hep G2 hepatocellular cells, to express NKp44L (Supplementary Figure 4), suggests that NKp44^+^ NK cells do not target HSCs.

## DISCUSSION

This study provides insights into the role of intrahepatic NK cells in chronic HCV infection. While it has consistently been reported that peripheral blood NK cells are altered in chronically HCV-infected patients, compared with healthy controls,^1,3–5^ peripheral blood and intrahepatic NK cells behave differently. One of the most intriguing finding of this study is the specific and significant accumulation of intrahepatic NKp44^+^ NK cells, which is correlated with both viral load and liver fibrosis in HCV-infected patients. This accumulation of NKp44^+^ NK cells has not been observed in vivo in either chronic HBV^+^ or NASH patients; it has been described, rarely, in certain situations, including in a subsets of RORγt^+^ innate lymphoid cells (ILCs)^24,32,33^ and NK cells in the decidua,^34^ and in peripheral blood NK cells after infection by the chikungunya virus^35^ and by HIV-1.^36^ The data we present here are consistent with the hypothesis that NKp44^+^ NK cells might have deleterious effects in tissue of patients with chronic HCV infection, as they have previously been suggested to do in the intestinal mucosa of patients with Crohn disease.^37^ This contrasts with the protective roles played by the other NCRs in HCV, such as the inverse correlation between NKp46^bright^ NK cells and the severity of liver inflammation,^36,38^ and the protection against HCV infection mediated by NKp30.^39^ The antagonistic roles of NCRs might be explained by the constitutive expression of NKp30 and NKp46 on NK cells, compared with the strict association of the cell-surface induction of NKp44 with cell activation.^9,10^ This point also highlights the key role of cell activation in the development of the liver fibrosis, as described.^40^

Our in-depth functional analysis of intrahepatic NK cells from HCV-infected patients revealed that expression of IFN-γ was inversely associated with the frequency of NKp44^+^ NK cells, whereas NKp46^high^ NK cells secrete larger amounts of IFN-γ than NKp46^dim^ NK cells.^37^ These data are consistent with the antifibrotic function of IFN-γ-producing NK cells mediated by the inhibition of HSCs in a TNF-related apoptosis-inducing ligand and NKG2D-dependent manner.^41^ We demonstrated, however, that TNF-α production was mainly confined to the intrahepatic NKp44^+^ NK cells, as previously reported for RORγt^+^ ILC, which use the selective expression of TNF-α by NKp44 to sense the environment.^26^ It has also been shown that hepatic dendritic cells acquire the marked ability to stimulate NK cells TNF-α-dependently in liver fibrosis.^42^ In view of these results, it was striking that the serum level of TNF-α correlated significantly with the fibrosis stage, as seen in previous studies of chronic HCV.^29,43^ As TNF-α is a major proinflammatory cytokine involved in several chronic diseases, the in vivo role of NKp44^+^ NK-derived TNF-α certainly requires further investigation in other associated pathologies.

We can thus hypothesize that high expression of TNF-α by intrahepatic NKp44^+^ NK cells might result from direct contact with HCV-infected hepatocytes, as previously shown in vitro.^44^ Nonetheless, the expression of NKp44 ligands on hepatocytes must be explored to illuminate more clearly the physiological relevance of NKp44 engagement in the intrahepatic NK cells of HCV-infected patients. It is important to note that NKp44L is expressed on Hep G2 hepatocyte cells, but not on LX2 HSCs. We propose that recognition of NKp44 by its ligand on intrahepatic NK cells from HCV patients might play a role in the TNF-α-dependent destruction of hepatocytes, while protecting HSCs. This TNF-α-dependent mechanism could lead to faster progression of liver fibrosis and cirrhosis, which are the main causes of morbidity and mortality in patients with HCV infection.^45^

Together with the previously described specific localization of NK cells to necrotic areas in liver biopsy specimens of HCV-infected patients,^25^ our data suggest that intrahepatic TNF-α-producing NKp44^+^ NK cells play a deleterious role in immune-mediated liver injury during chronic infection. Hence, these data deepen our understanding of liver immunology in fibro-inflammatory disease and suggest that an approach targeting NKp44 could present an attractive therapeutic strategy in HCV infection and potentially in other chronic inflammatory diseases.

## Supplementary Material

Supplemental Digital Content
