# Supplementary material for: Accumulation of Intrahepatic TNF-α-Producing NKp44+ NK Cells Correlates With Liver Fibrosis and Viral Load in Chronic HCV Infection
Source: Medicine (Baltimore). 2016 May 13;95(19):e3678. doi: 10.1097/MD.0000000000003678 (PMC4902546; doi:10.1097/MD.0000000000003678)

**Supplementary FIGURE 1. Gating strategy for NKp44^+^ NK cells.** CD3^-^CD56^+^ NK cells from peripheral blood (PB) and liver biopsies of a representative HCV^+^ sample were gated within the CD45^+^ lymphocytic population. NK cells positive for NKp44 were detected with a specific anti-NKp44-PE monoclonal antibody.


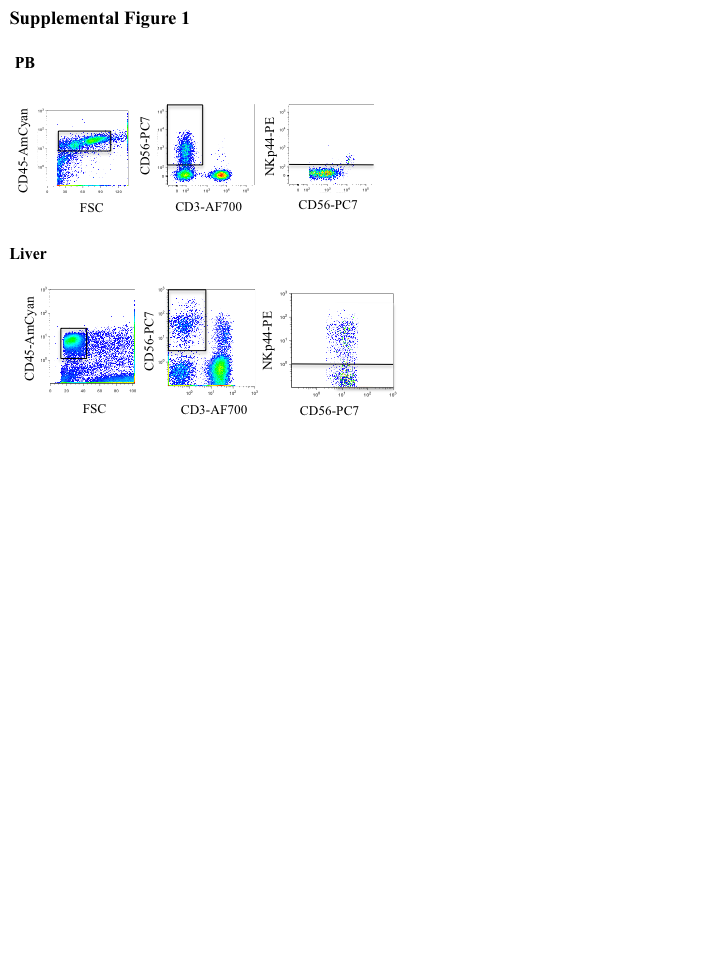


**Supplementary FIGURE 2. Expression of NK cell markers on intrahepatic NK cells.** Frequency of CD16, NKp46, NKp30, panKIR-L (KIR2DL1, KIR2DL2/DL3, and KIR3DL1), NKG2A, NKG2C, CD62L, and Cx3CR1 in peripheral blood (PB) and intrahepatic (Liver) CD3^-^CD56^+^ NK cell from the 21 HCV-infected patients. Statistical analysis used the Wilcoxon rank test; **: p<.01; ***: p<.001.

**
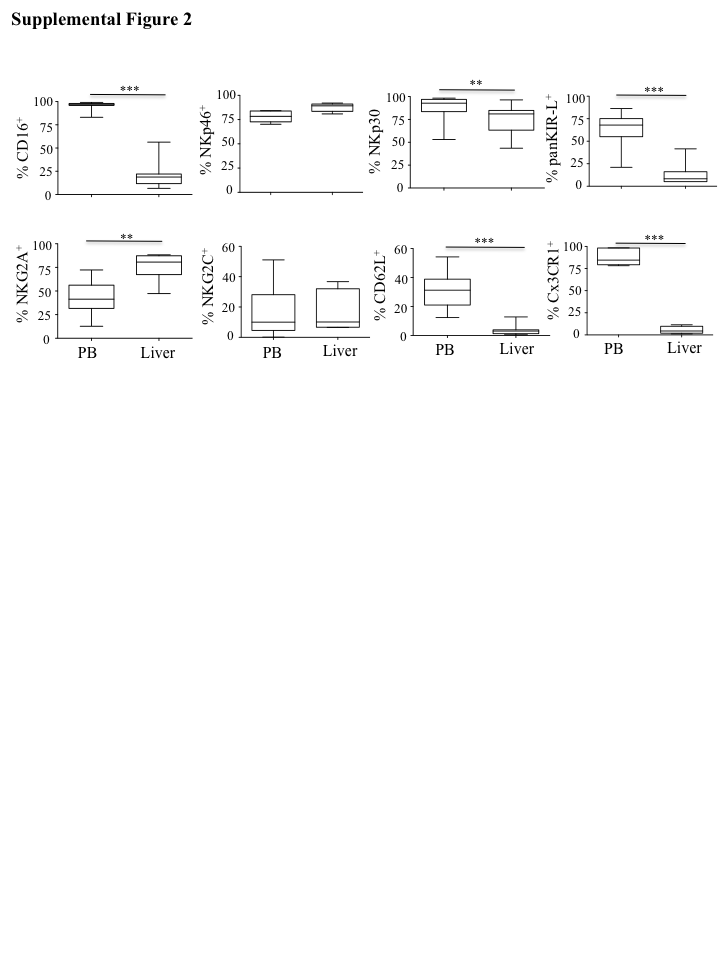
**

**Supplementary FIGURE 3. Linear regression between the frequency of peripheral or intrahepatic NK cells and the cytokine levels in the plasma of the 21 HCV^+^ samples**. The Spearman test showed significant statistical correlations.

**
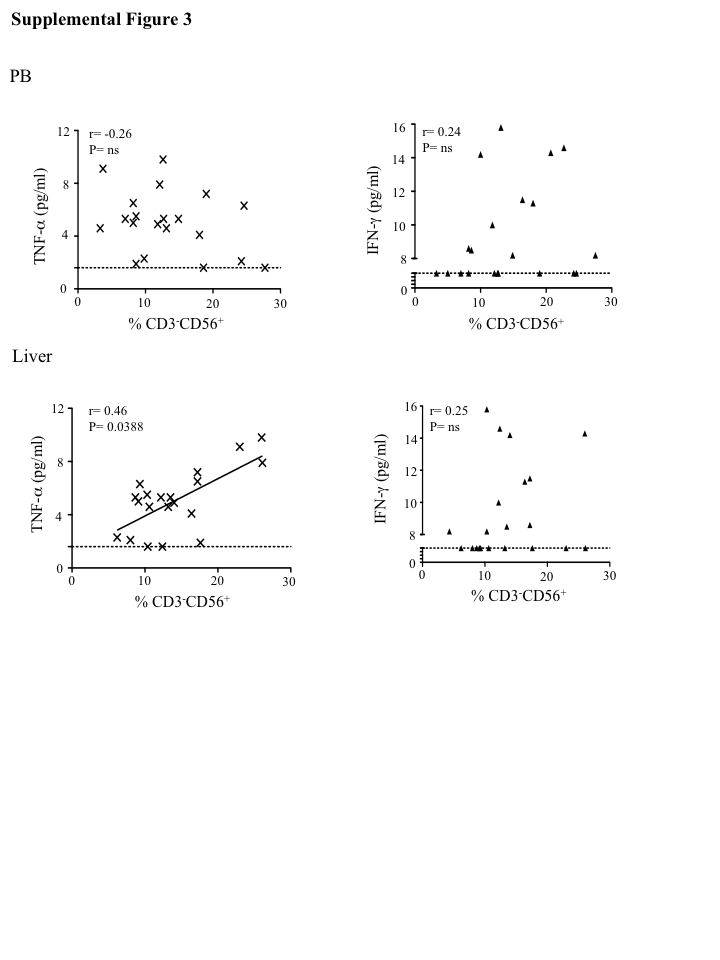
**

**Supplementary FIGURE 4. Cell-surface expression of NKp44 ligands**. Analysis was assessed by flow cytometry. The indicated cell lines were stained with anti-NKp44L mAb (bold lines), NKp44-Ig fusion protein (R&D systems) (bold lines), or with their respective controls (gray histograms), as described.^11^ Data are representative of at least 3 independent experiments.


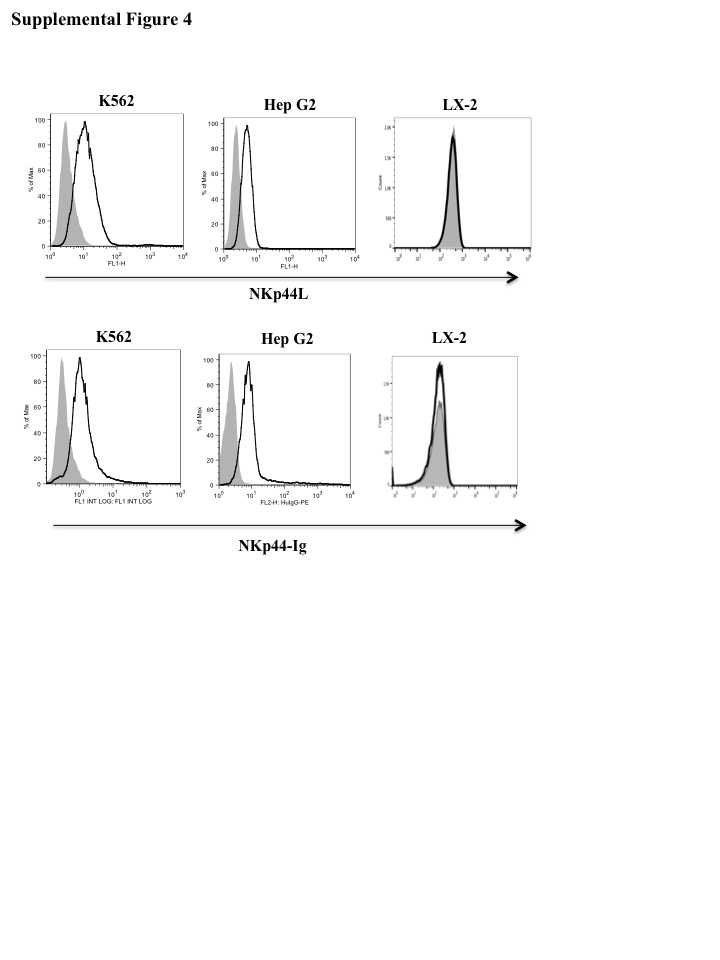

Supplement: Supplemental Digital Content [file medi-95-e3678-s001.doc]
